# Nineteen-Year Evidence on Measles–Mumps–Rubella Immunization in Mexico: Programmatic Lessons and Policy Implications

**DOI:** 10.3390/vaccines13111126

**Published:** 2025-10-31

**Authors:** Rodrigo Romero-Feregrino, Raul Romero-Feregrino, Raul Romero-Cabello, Berenice Muñoz-Cordero, Benjamin Madrigal-Alonso, Valeria Magali Rocha-Rocha

**Affiliations:** 1 Asociación Mexicana de Vacunología, Mexico City 06760, Mexico; 2 Instituto para el Desarrollo Integral de la Salud (IDISA), Mexico City 06700, Mexico; 3 Technical Council of the Employers’ Sector CONCAMIN, Instituto Mexicano del Seguro Social (IMSS), Mexico City 06600, Mexico; 4 Department of Infectology, UMAE Hospital de Pediatria “Dr Silvestre Frenk Freund” Centro Médico Nacional Siglo XXI Instituto Mexicano del Seguro Social (IMSS), Mexico City 06720, Mexico; 5 Department of Infectology, Hospital General de México Dr. Eduardo Liceaga, Mexico City 06720, Mexico; 6 Department of Microbiology and Parasitology, Universidad Nacional Autónoma de México (UNAM), Mexico City 04360, Mexico; 7 Pediatrics Department, Hospital General IMSS-Bienestar de Cuajimalpa, Mexico City 05500, Mexico; 8 School of Life and Health Sciences, Universidad Popular Autónoma del Estado de Puebla, Puebla 72410, Mexico

**Keywords:** measles–mumps–rubella vaccine, MMR, immunization programs, vaccination coverage, Mexico, catch-up immunization, vaccine supply and distribution, measles/epidemiology, rubella/prevention and control, vaccine data quality, MMR vaccine, immunization program evaluation

## Abstract

Background: In Mexico, the measles vaccine was first introduced in 1971. The last case of measles acquired through endemic transmission was recorded in 1995. In 1998, the monovalent measles vaccine was replaced by the combined measles–mumps–rubella (MMR) vaccine. The MMR vaccination schedule consists of two doses: the first is administered at 12 months of age, and the second is administered at either 18 months or 6 years of age. Materials and Methods: A retrospective analysis was conducted using secondary data from 2006 to 2024. Vaccine procurement and administration records from IMSS, ISSSTE, and SSA were reviewed to evaluate the performance of both the MMR and MR programs, focusing particularly on the trends in coverage and data consistency across institutions. Results: The analysis revealed persistent inconsistencies between vaccine procurement and administration for both the MMR and MR vaccines across all institutions. Several years exhibited notable mismatches, including surpluses and deficits in the administered doses relative to their procurement. Between 2006 and 2024, only 69 million of the 91.6 million required MMR doses were administered in Mexico, leaving a deficit of approximately 22.5 million doses (25% of the target population). For MR, a cumulative deficit of approximately 24.6 million procured but unadministered doses was identified. National coverage remained suboptimal, with significant variability across years and institutions. Comparisons with WHO and ENSANUT data indicated marked discrepancies. The seroprevalence findings, along with the 2025 measles outbreak, confirm significant gaps in immunity. Discussion: This study highlights systemic challenges in Mexico’s MMR vaccination program, including inconsistencies in vaccine procurement, administration, and reported coverage across institutions. Overestimated official MMR coverage rates and unclear target definitions for MR contribute to program inefficiencies and missed vaccination opportunities. The resurgence of measles in 2025, along with persistently high incidences of mumps, aligns with the observed immunity gaps, although a direct causal relationship cannot be established from this study. These findings are consistent with previous national studies and seroprevalence data. Conclusions: Despite limitations in the data, this study effectively evaluated the performance of Mexico’s MMR vaccination program, identifying critical gaps in coverage, data reliability, and operational alignment. The findings underscore the need for improved procurement planning, harmonized coverage estimates, and robust monitoring systems. To address the existing gaps in immunity, catch-up campaigns should prioritize the use of the MMR vaccine over MR. Strengthening nominal coverage tracking and implementing evidence-based strategies are essential to restoring public trust and maintaining the goals of measles elimination.

## 1. Introduction

The first measles vaccine was licensed for public use in the United States in 1963 [1], followed by the mumps vaccine in 1967 [2] and the rubella vaccine in 1969. In 1971, a combined measles, mumps, and rubella (MMR) vaccine was also licensed [3].

In Mexico, the measles vaccine was introduced in 1971 using the Schwarz strain, followed by the Edmonston–Zagreb strain. A measles epidemic during the 1989–1990 period led to 8150 deaths and 89,163 reported cases. The last case of measles acquired through endemic transmission in Mexico was recorded in 1995. In 1998, the monovalent measles vaccine was replaced with the combined MMR vaccine. Additionally, in 2001, measles vaccination was extended to individuals aged 13 years and older using the measles-rubella (MR) vaccine [4]. The overarching goal of Mexico’s MMR vaccination program is to sustain high and equitable coverage to prevent outbreaks and maintain the elimination of measles and rubella, in alignment with regional elimination goals [5].

In Mexico, the MMR vaccination schedule consists of two doses. The first dose is recommended at 12 months of age; if it is not administered at that time, it should be given at the earliest opportunity. Since 2022, the second dose has been administered at 18 months of age. For cohorts vaccinated prior to 2021, the second dose should be provided at 6 years of age. Furthermore, the measles–rubella (MR) vaccine is recommended for individuals aged 10 years and older who have no vaccination history, an incomplete vaccination schedule, or an unknown vaccination status [4].

The Americas became the first region in the world to achieve the elimination of measles in 2016 [5]. In Mexico, endemic measles transmission was interrupted in 1996. Following a rigorous verification process, the Pan American Health Organization officially recognized the regional elimination of measles in 2016 [6].

Reports from the World Health Organization (WHO) [7] and the National Health and Nutrition Survey (ENSANUT) [8,9,10,11,12], conducted by the Ministry of Health of Mexico, provide detailed information on vaccination coverage in Mexico, as shown in Table 1.

Table 2 summarizes newly reported cases of rubella, mumps, and measles in Mexico.

Evaluations of Mexico’s National Immunization Program, particularly regarding influenza [15] and Bacillus Calmette–Guérin (BCG) vaccines [16], have uncovered inconsistencies in the official datasets that indicate systemic weaknesses. Key challenges include difficulties in accurately defining the target populations, disruptions in the supply chain, operational inefficiencies, and deficiencies in data quality and reporting. In light of declining vaccination coverage rates, recurring vaccine shortages, and the known structural limitations of the Mexican healthcare system, we conducted a thorough review of the official records related to vaccine procurement, administration, and coverage.

This study aims to describe and critically analyze the available data to clarify the current performance of the program and provide actionable recommendations.

Our focus is exclusively on institutional and programmatic aspects of vaccine procurement, administration, and reporting. We do not address behavioral factors such as vaccine hesitancy or refusal. Due to the variability and inconsistencies across different data sources, we expected to find a low level of agreement between official figures and our independent estimates. The findings are intended to support the urgent development of evidence-based strategies to strengthen the national immunization system.

## 2. Material and Methods

Study Design. This study was a retrospective, longitudinal, and ecological analysis conducted using secondary data obtained from official government repositories. The study period spans 19 years, from 2006 to 2024.

Data Sources. Data were gathered from Mexico’s three main public health institutions: the Mexican Social Security Institute (Instituto Mexicano del Seguro Social, IMSS, Mexico), the Institute for Social Security and Services for State Workers (Instituto de Seguridad y Servicios Sociales de los Trabajadores del Estado, ISSSTE, Mexico), and the Ministry of Health (Secretaría de Salud, SSA, Mexico). Together, these entities provide healthcare services to approximately 98% of the national population, corresponding to over 130 million individuals as of 2023, which includes an estimated 2.1 million live births per year [17,18,19,20].

Variables and Data Collection. The primary variables examined in this study included the number of vaccine doses procured, the number of doses administered, and the vaccination coverage rates. Data were obtained from publicly accessible institutional reports, annual health statistics, and official registries. To ensure the quality and reliability of the data, only validated datasets issued by government authorities were used. Additionally, specific data requests were made through the National Institute for Transparency, Access to Information, and Protection of Personal Data (Instituto Nacional de Transparencia, Acceso a la Información, y Protección de Datos Personales, INAI. Mexico City, Mexico).

Analytical Approach. Descriptive and comparative analyses were performed to investigate the trends over time in vaccine procurement and administration. These data were utilized to develop theoretical models intended to explain the observed dynamics. The modeling process helped to identify operational gaps and inefficiencies within the national immunization program. A linear regression ANOVA was performed to compare vaccination coverage values across three institutions, adjusting for year.

Ethical Considerations. This study solely relied on secondary data sources that were publicly available and devoid of any personal identifiers. In accordance with national and international ethical standards for research involving non-identified datasets, ethical review and informed consent were not required.

Data Requirements and Sources. Key data elements essential for this analysis included the number of individuals covered by each institution, detailed vaccine-specific information (such as vaccine type and indication), and the corresponding numbers of doses acquired and administered.

Data concerning vaccine procurement were obtained through formal information requests submitted to INAI and subsequently retrieved from the Institute’s official databases [21,22,23,24,25,26,27,28,29,30,31,32,33,34,35,36,37,38]. Records of vaccine administration were accessed from the historical data archives maintained by the IMSS [39], ISSSTE [40], and SSA [41]. Population estimates were sourced from official projections provided by the National Population Council (Consejo Nacional de Población, CONAPO. Mexico City, Mexico) [19,20].

Data Processing and Indicator Definitions. After acquiring the data, analytical frameworks were established to facilitate comparative evaluations across healthcare institutions and over time. A set of core indicators was defined to aid in data interpretation:

- Theoretical Target Population: the estimated number of individuals eligible for vaccination, calculated based on each vaccine’s specific indications and the population assigned to the corresponding healthcare institution.
- Annual Procurement and Year-on-Year Change: total number of vaccine doses procured each year, along with the percentage change compared to the previous year.
- PUR (Procurement-to-Target Ratio): the proportion of vaccine doses acquired relative to the theoretical target population.
- APP (Application-to-Procurement Ratio): the proportion of administered doses compared to the total number of doses acquired.
- COV (Coverage Rate): the proportion of administered doses in relation to the theoretical target population.

The estimated target population was calculated by aligning demographic projections from CONAPO with eligibility criteria defined in national immunization guidelines.

Regarding MMR (measles, mumps, and rubella) vaccination, Mexican national guidelines have traditionally recommended a two-dose schedule: the first dose at 12 months of age and the second at 6 years. As of 2021, this schedule was updated for individuals born in that year and later, with the second dose now recommended at 18 months. For individuals born prior to 2021, the second dose remains scheduled for 6 years of age [4]. These data are presented in Table 3.

For each healthcare institution, we estimated the theoretical target population using a formula that combined the total national target population with the coverage proportion provided by each respective provider (IMSS, ISSSTE, and SSA). These coverage proportions were based on the number of beneficiaries reported by each institution and were used to appropriately allocate the national target population. The distribution percentages of the beneficiary population across institutions are shown in Table 4.

The target population estimates specific to each institution were calculated using demographic data and the following methodology:

- Total theoretical target population: the combined number of individuals aged 12 months and 6 years for all relevant years, along with those aged 18 months for cohorts born in 2021 and later, following MMR vaccination guidelines.
- IMSS: total theoretical target population × annual proportion of the population affiliated with IMSS.
- ISSSTE: total theoretical target population × annual proportion of the population affiliated with ISSSTE.
- SSA: total theoretical target population × annual proportion of the population covered by SSA.

Three key indicators were used to evaluate the institutional performance in vaccine procurement and administration:

- %PUR (Procurement-to-Target Ratio): (Doses acquired/Theoretical target population) × 100
- %APP (Application-to-Procurement Ratio): (Doses administered/Doses acquired) × 100
- %COV (Coverage Rate): (Doses administered/Theoretical target population) × 100

Additionally, information was requested regarding the measles–rubella (MR) vaccine. According to the national immunization program, this vaccine should be administered to individuals aged 10–13 years and older, depending on the specific year, in cases of prior non-vaccination, incomplete vaccine schedules, or unknown vaccination status. However, a theoretical target population for MR was not defined due to the absence of clear guidelines on who exactly should receive the vaccine. Therefore, only the available data from the institutional sources are discussed and compared.

Moreover, the number of unvaccinated individuals was estimated for each institution and in total by subtracting the number of doses administered from the corresponding theoretical target population. The resulting coverage rates (%COV) were then compared against the benchmark targets established by the World Health Organization (WHO) [7] and ENSANUT.

All data processing and statistical analyses were conducted using Microsoft Excel, and graphs and visualizations were created by integrating selected variables, ensuring that only the most representative data were included in the final presentation.

Missing procurement data were excluded from the main analyses to avoid distorting the relationships between procurement, application, and coverage. When data for certain years or institutions were not publicly available, the corresponding values were treated as missing rather than zero. This approach prevents artificial inflation or deflation of institutional performance indicators. However, it also implies that years with unavailable procurement data may underestimate true acquisition volumes.

## 3. Results

Table 5 displays the number of years for which vaccine procurement data were available for each institution and vaccine over a maximum period of 19 years (2006–2024). Completeness is calculated as the percentage of available years relative to the total possible years. It is important to note that the absence of data for certain years may indicate that no vaccines were procured during those periods. However, it could also reflect a lack of publicly available information or incomplete reporting.

The source data on MMR and MR vaccine procurement were incomplete for each institution; therefore, the total number of doses purchased across the three institutions was not used for analysis. Instead, procurement data were analyzed separately for each institution.

For MMR, the data from the SSA were missing in 2007, 2008, 2010, 2013–2017, 2021, and 2022. For IMSS, data were unavailable from 2006 to 2013, and for ISSSTE, the missing years were 2009, 2012, 2015, and 2017. In the case of MR, the SSA lacked data for 2021 and 2022; IMSS was missing data from 2006 to 2011; and ISSSTE had gaps for 2012, 2015, 2017, and 2022.

**Figure 1:**
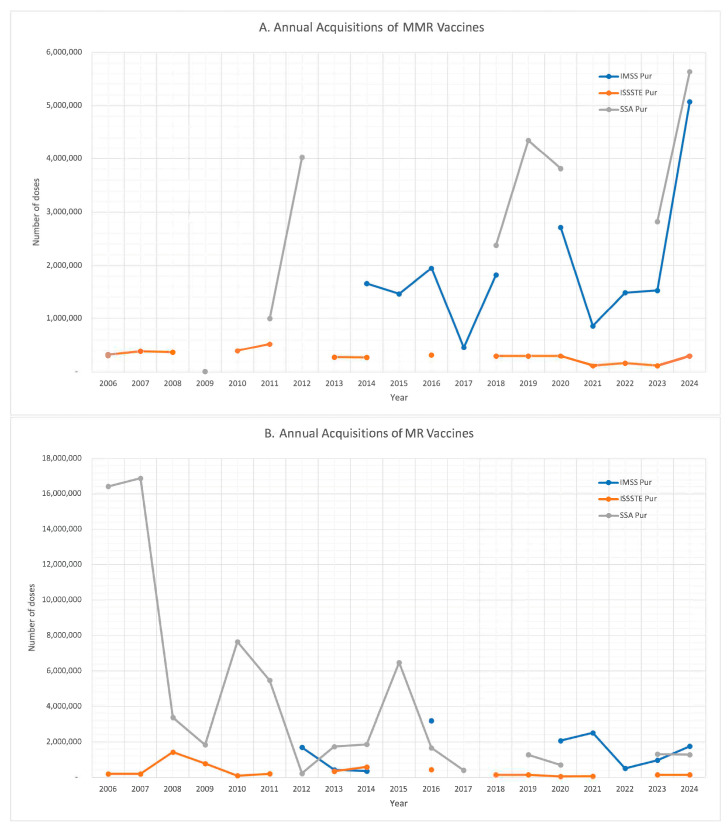
Annual acquisitions of vaccines: (**A**). MMR; (**B**). MR.

Figure 1 presents the annual number of MMR and MR vaccine doses procured by each institution. A comparative analysis was conducted across institutions for each year, revealing significant year-to-year variation in procurement volumes. These fluctuations likely reflect changes in institutional procurement practices and shifting programmatic priorities. In some instances, it remains unclear whether the missing data indicate a true absence of procurement (i.e., zero doses purchased) or simply unreported or unavailable information.

**Figure 2:**
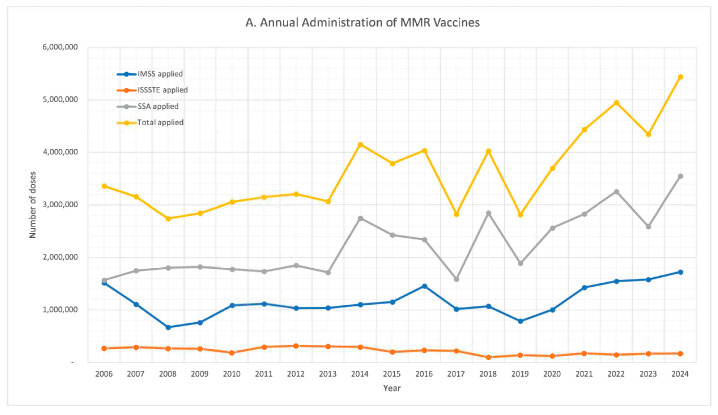

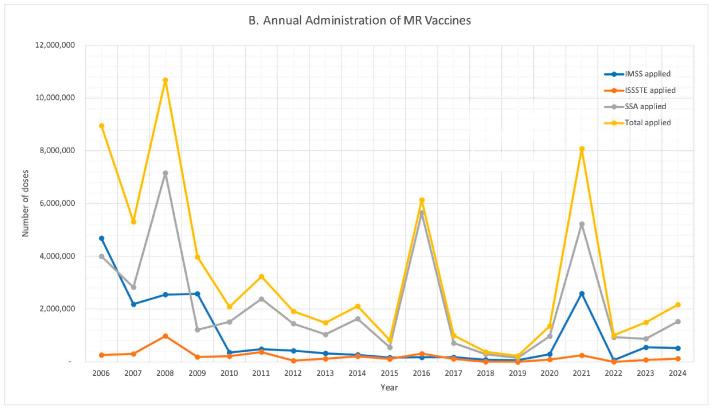
Annual administration of vaccines: (**A**). MMR; (**B**). MR.

All three institutions provided consistent data on the administration of the MMR and MR vaccines throughout the entire study period (see Figure 2).

Figure 2 illustrates significant fluctuations from year to year in the administration of MMR and MR vaccine doses, which were evident both at the institutional level and in the aggregated national data.

**Figure 3:**
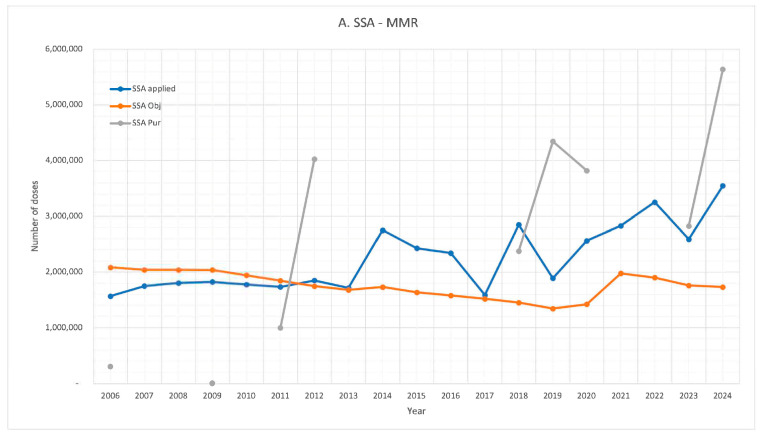

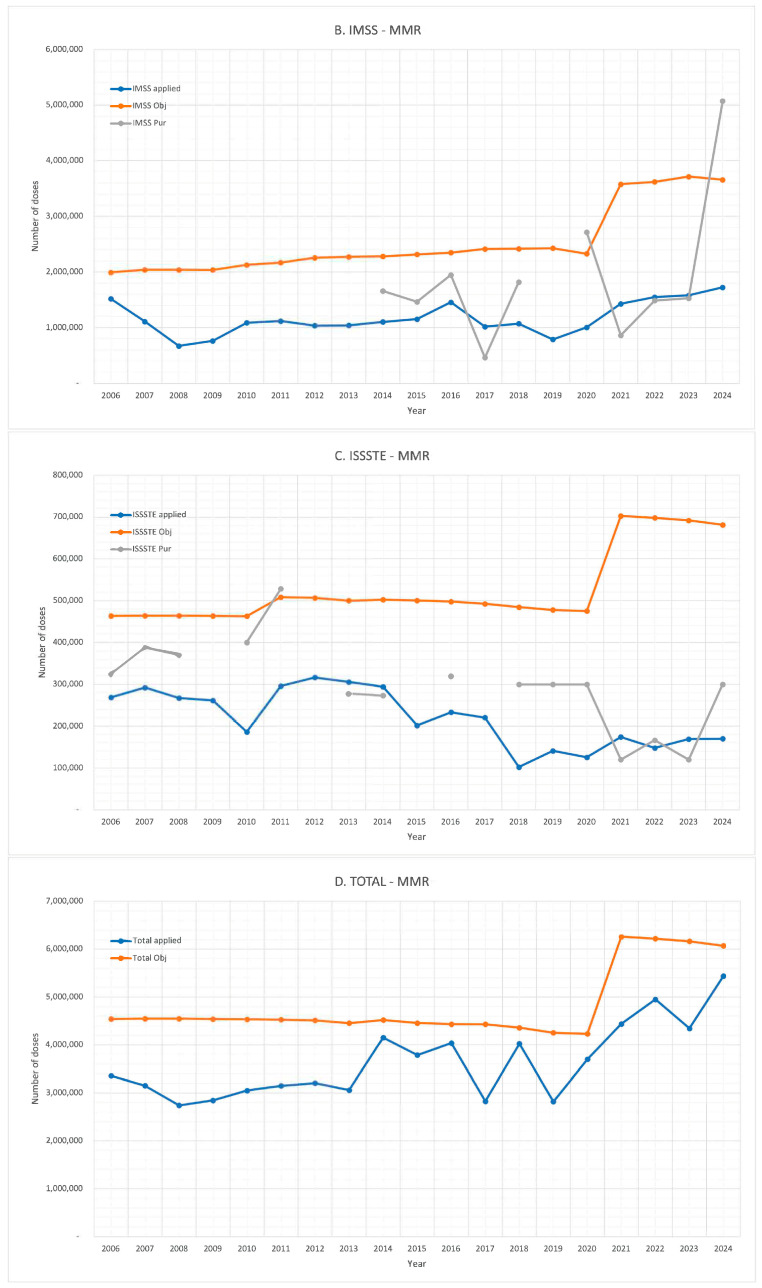
MMR vaccines: comparison of vaccine acquisitions, theoretical target population, and number of doses administered. (**A**). SSA, (**B**). IMSS, (**C**). ISSSTE, (**D**). total.

Figure 3 provides a comparative analysis of MMR vaccine procurement (PUR), the theoretical target population (OBJ), and the doses administered (Applied) across the three main healthcare institutions.

From 2014 to 2024, the number of doses administered by the SSA frequently exceeded the theoretical target population in most years. In contrast, both IMSS and ISSSTE consistently reported lower administration levels than their respective target populations. When considering all three institutions combined, the total number of doses administered remained below the estimated target population.

Among the institutions with available procurement data, the vaccine purchases displayed considerable variability from year to year, rather than a consistent annual quantity. Additionally, all institutions reported years when the number of doses procured exceeded those administered, as well as years when procurement fell below administration levels. Overall, the SSA tends to procure more doses than its theoretical target population, while the IMSS and ISSSTE generally acquire fewer doses than needed. Furthermore, in many years, the number of doses procured significantly exceeded the number administered across all institutions.

**Figure 4:**
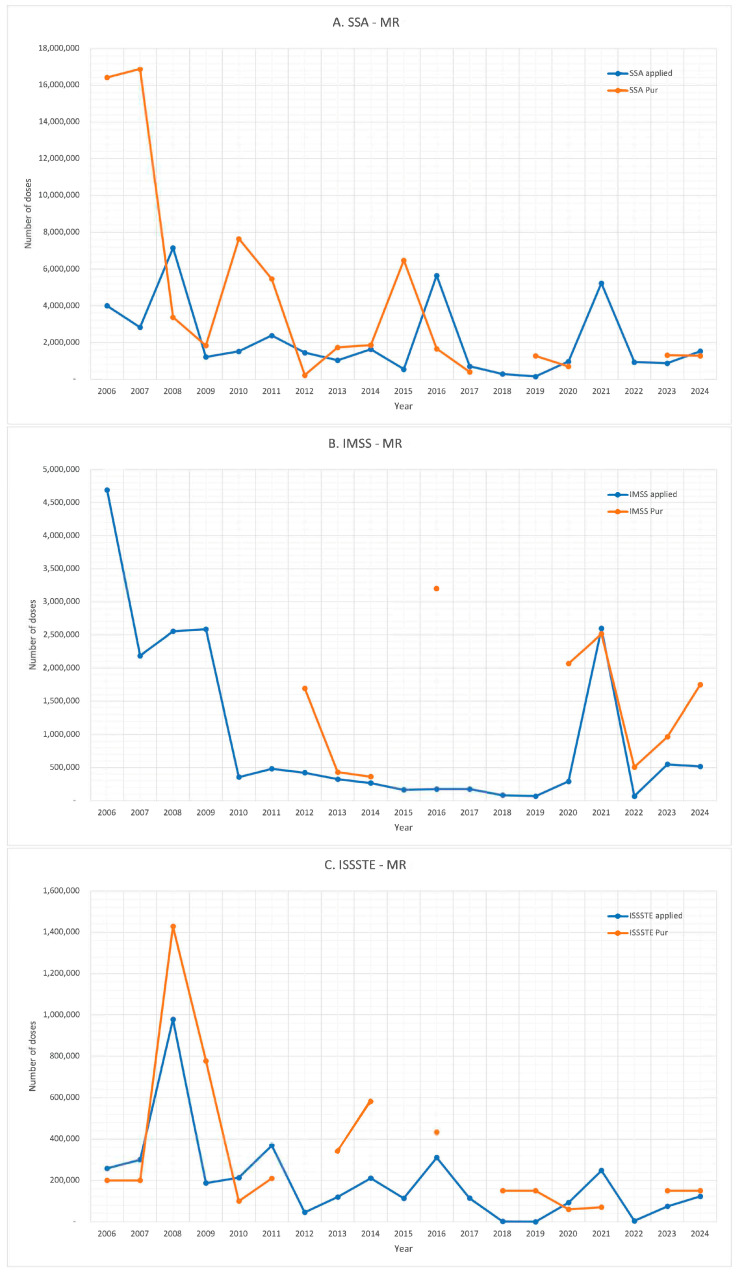

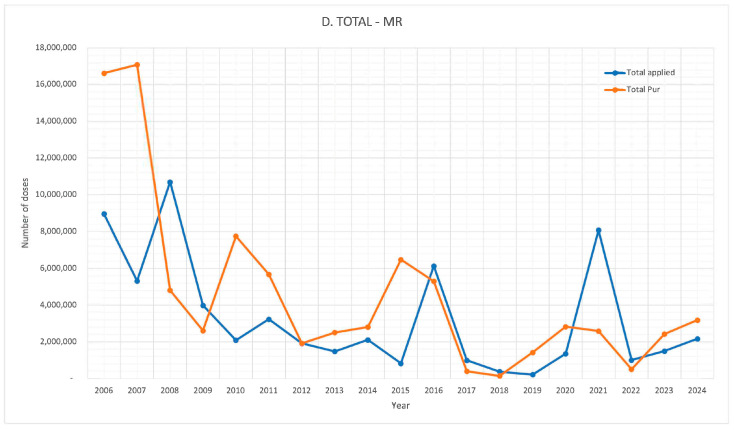
MR vaccines: comparison of vaccine acquisitions and number of doses administered. (**A**). SSA, (**B**). IMSS, (**C**). ISSSTE, (**D**). total.

Although procurement data are missing for certain years, Figure 4 shows the general trend of the relationship between the number of MR vaccine doses procured and those administered. However, some years show significant discrepancies. For the SSA, significantly higher procurement than administration was noted in 2006, 2007, 2010, and 2015. In the case of IMSS, the discrepancies occurred in 2012, 2016, and 2020. For ISSSTE, in 2008, 2009, 2013, and 2014, there were higher numbers of doses purchased than administered.

Conversely, more doses were administered than procured in 2004, 2012, and 2016, for SSA, and 2015, 2017, and 2021, for ISSSTE. These patterns reflect inconsistencies in the procurement and delivery cycles across the three institutions.

Between 2006 and 2024, a total of 87,103,897 doses of the MR vaccine were reported as procured, while 62,453,264 doses were administered. This leaves 24,650,633 doses that were not used, approximately 28% of the procured doses.

**Figure 5:**
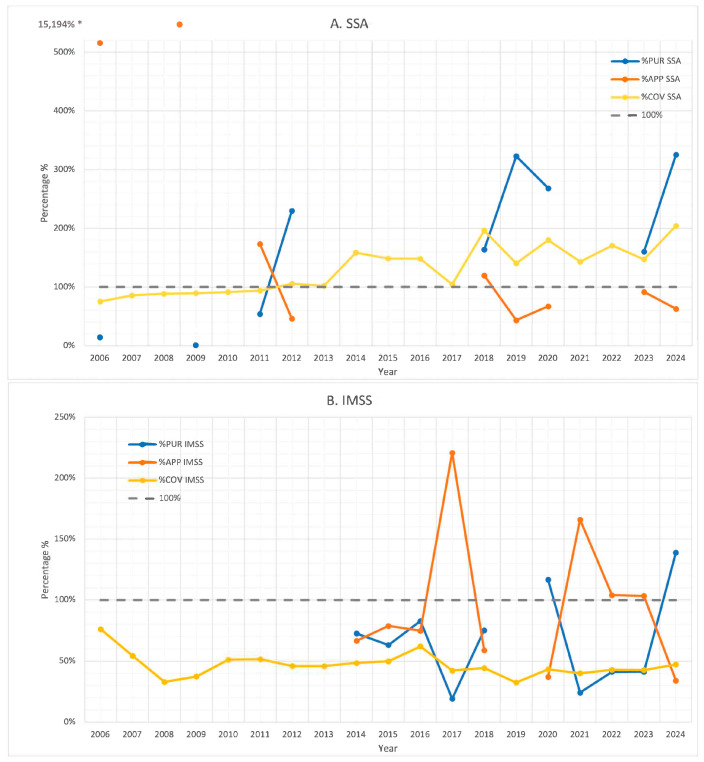

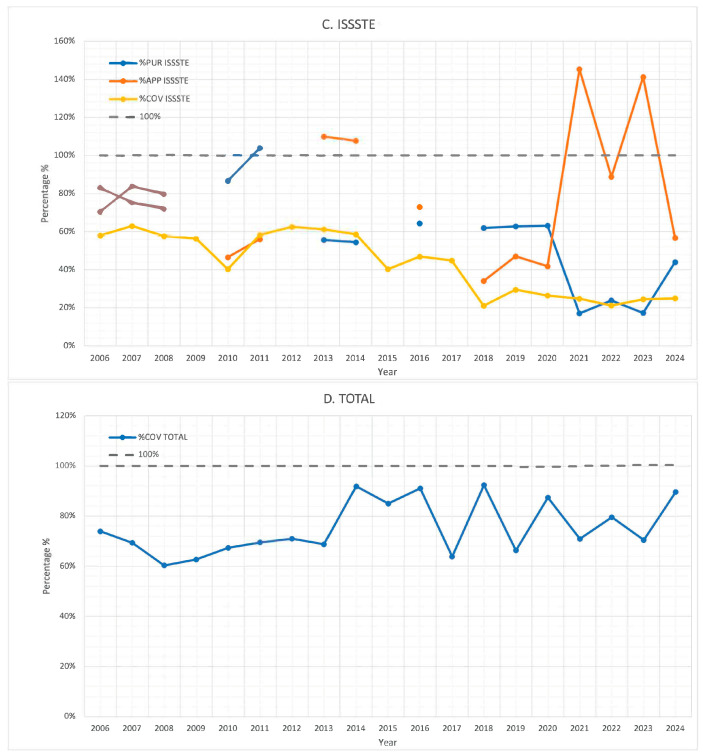
MMR vaccine indicators PUR, APP, and COV. (**A**). SSA, (**B**). IMSS, (**C**). ISSSTE, (**D**). total. * The outlier value for 5A.SSA in 2009 (15,194%) is visually truncated for readability.

Figure 5 illustrates the calculated percentages for the procurement-to-target ratio (%PUR), application-to-procurement ratio (%APP), and coverage rate (%COV) for each institution throughout the study period.

For SSA, %PUR values were consistently high in the years after 2012, ranging from 161% to a peak of 325% in 2024. In contrast, prior to 2012, %PUR remained low, fluctuating between 1% and 54%. The %APP indicator during the years with available procurement data showed substantial variability, ranging from 44% to an extreme of 15,184%, with four years surpassing 100%. Two extreme outliers (2006: 516% and 2009: 15,194%) are illustrated outside the graph’s proportional scale to maintain the readability of other data points. Similarly, %COV ranged from 75% to 204% during the study period and consistently exceeded 100% in all years after 2012.

For IMSS, the %PUR indicator in years with available procurement data after 2014 remained generally low, ranging from 19% to 83%, with two years (2021 and 2024) exceeding 100% (117% and 139%). During the same period, the %APP showed a mixed pattern: in six years, it remained below 100% (ranging from 34% to 79%), while in four years, it exceeded 100%, reaching values between 103% and 221%. The %COV indicator remained consistently low throughout the study period, staying at or below 51% in all years except for one, in which it reached 76%(2006).

In the case of ISSSTE, %PUR generally remained values ranged from 17% to 86% with only one year surpassing the 100% threshold. The %APP displayed significant variability, ranging from 34% to 145%. Four years recorded values above 100%, suggesting that more doses were administered than officially procured. The %COV indicator remained below 63% throughout the period, with values ranging from 21% to 63%.

When aggregating data from all three institutions, only the %COV indicator was calculated due to incomplete procurement data. Across the combined institutions, %COV ranged from 60% in 2008 to 92% in 2018, and in all years, it remained below the 100% threshold.

A linear regression ANOVA was performed to compare the vaccination coverage values across the three institutions while accounting for the year. This approach was preferred over a one-way ANOVA to address potential temporal trends. The overall model was found to be statistically significant (*p* < 0.001). After adjustment, the institution showed a significant effect (*p* < 0.0001), indicating differences in vaccination coverage values between the institutions. The year was not statistically significant (*p* = 0.2196). These results suggest that there are disparities in vaccination coverage values among institutions, independent of the year.

**Figure 6:**
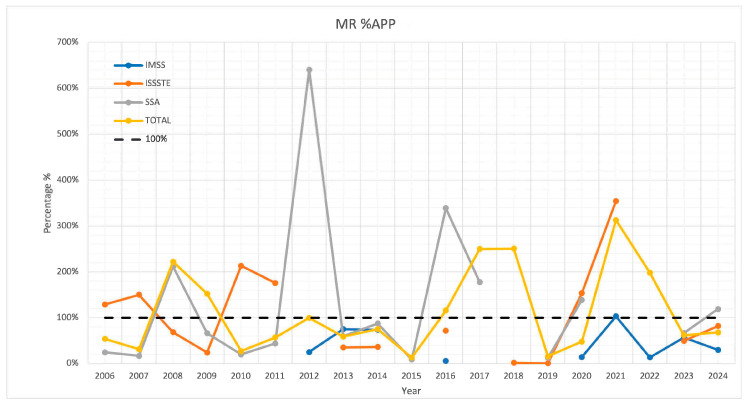
APP indicator per institution and total for MR vaccines.

Figure 6 illustrates the MR vaccine application-to-procurement ratio (%APP) for each of the three institutions, as well as the combined total. This indicator measures the proportion of vaccine doses administered relative to those procured.

For SSA, %APP exceeded 100% in six years, with values ranging from 139% to 640%. However, in ten years, it remained below 100%, with values between 9% and 88%. In the case of IMSS, only one year displayed a %APP above 100% (at 103%), while the other years ranged from 5% to 75%. For ISSSTE, nine years recorded values below 100%, ranging from 0.2% to 82%, while six years exceeded 100%, with values between 129% and 354%.

For the aggregated total across all institutions, data from all 19 years were included. In years with missing information from a specific institution, it was assumed that both the procurement and administration were zero. Following this method, 58% of the years showed %APP values below 100%, ranging from 13% to 75%. In contrast, 42% of the years exceeded 100%, with values between 116% and 313%.

**Figure 7:**
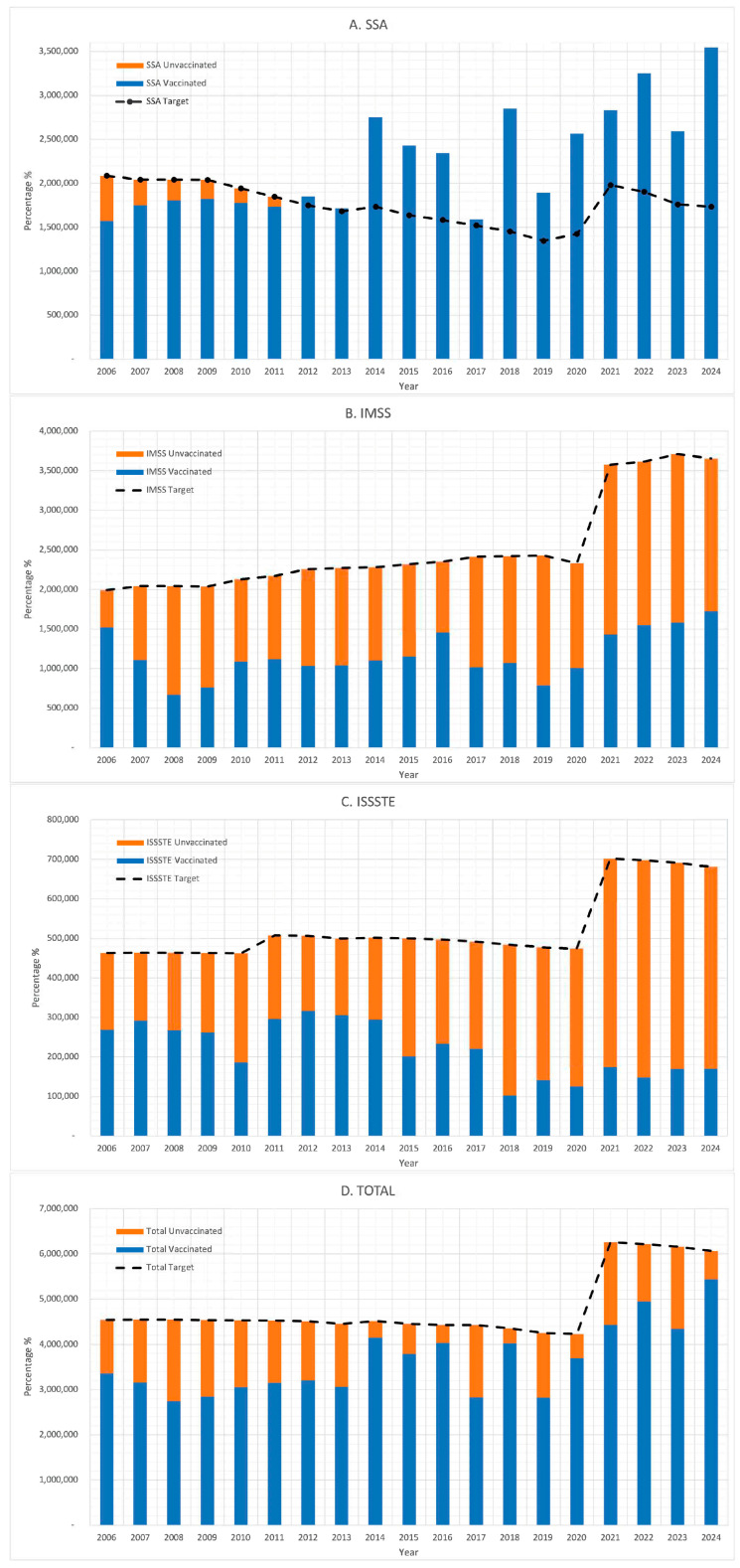
MMR vaccine coverage and proportion of unvaccinated, 2006–2023. (**A**). SSA, (**B**). IMSS, (**C**). ISSSTE, (**D**). Total.

Figure 7 shows that unvaccinated populations were present in all three institutions throughout the study period. From 2006 to 2024, the estimated theoretical target population for MMR vaccination required 91,590,106 doses. However, only 69,081,347 doses were administered, resulting in a deficit of 22,508,759 doses—representing 25% of the target population. By institution, IMSS and ISSSTE administered only 46% and 42% of the doses needed to meet their respective targets. In contrast, SSA administered 27% more doses than its calculated target.

**Figure 8:**
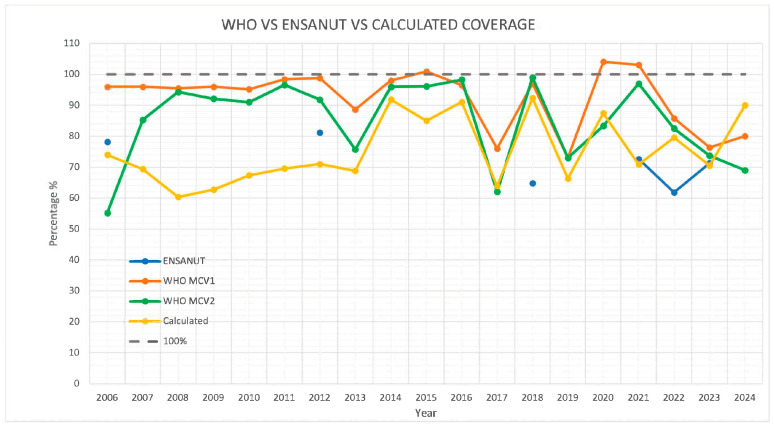
Comparison of WHO coverage of MC1 and MC2 vs. ENSANUT vs. Calculated coverage.

Figure 8 presents a comparison between the calculated coverage rates and those reported by the World Health Organization (WHO) for the measles-containing vaccine first dose (MCV1) and second dose (MCV2) [7]. Both datasets indicate a decline in coverage during 2020 to 2024. However, the magnitude of these changes differs substantially between the sources, suggesting potential variations in data collection methods or reporting criteria.

Figure 8 compares the WHO-reported coverage estimates for the first (MCV1) and second (MCV2) doses of the measles-containing vaccine [7] with the data from ENSANUT [8,9,10,11,12] and the calculated coverage from this study. While the WHO estimates for MCV1 and MCV2 consistently remain high—approaching or exceeding 90% in most years—the calculated coverage shows a notably lower trend throughout the period from 2006 to 2024. Discrepancies are especially evident in 2006–2013, 2015, 2021 and 2024. After 2019, the calculated values fall significantly below the WHO estimates.

## 4. Discussion

This study provides a comprehensive evaluation of the MMR vaccination program in Mexico, acknowledging significant limitations in data availability and quality—particularly with regard to vaccine procurement records. Despite these constraints, the integration of vaccine administration data and demographic estimates allowed for a robust assessment of national and institutional performance. By incorporating data from Mexico’s three major public health institutions (IMSS, ISSSTE, and the Ministry of Health), the analysis revealed heterogeneous patterns in coverage and operational consistency, as well as critical areas for programmatic improvement.

The methodology used in this study, although based on retrospective and administrative data, adheres to the principles of scientific inquiry in public healthcare systems research. The triangulation of institutional records, estimation of theoretical target populations, and use of standardized indicators support the validity and replicability of the analysis. Similar retrospective evaluations have been published in peer-reviewed journals to inform immunization policy and strengthen healthcare systems.

Although procurement data were limited in scope and completeness, the available information revealed significant irregularities in the annual acquisition of MMR and MR vaccines. These irregularities were characterized by abrupt and unexplained fluctuations from year to year, which are difficult to justify given the relative stability of the theoretical target population over time. Furthermore, the institutional records lacked sufficient detail to clarify the factors contributing to such variations.

In the case of MMR, procurement data were notably more incomplete than for MR, particularly within SSA. This may reflect differences in programmatic priorities—MR vaccines are often used in large-scale campaigns targeting adolescents and adults, which tend to be better documented—whereas MMR vaccines are part of routine pediatric immunization, potentially leading to less centralized tracking. Additionally, administrative restructuring and decentralization within SSA during the study period may have contributed to gaps in historical procurement records. Collectively, these irregularities highlight structural challenges in aligning vaccine logistics with service delivery and underscore the need to strengthen inventory management and data system

The absence of procurement data for specific years introduces potential bias in institutional comparisons. For instance, missing records could result in apparent under-procurement. Similarly, years without reported data might obscure true fluctuations in vaccine availability. These limitations highlight the urgent need for transparent, standardized reporting systems that consolidate procurement and administration data across institutions.

The analysis of vaccine administration relative to procurement identified marked inconsistencies across all institutions for both MMR and MR vaccines. In several years, the number of doses administered either significantly exceeded or fell below the number officially procured, suggesting a lack of alignment between supply and application. For MR, this discrepancy culminated in a deficit of approximately 24.6 million doses procured but not administered. For MMR, although procurement data were incomplete, similar patterns were observed. Potential explanations include the use of leftover stock from previous years, unreported doses, emergency purchases, lags in data reporting or overreporting especially in contexts where coverage targets are tied to institutional performance. However, the available records lacked sufficient granularity to verify these hypotheses. These inconsistencies highlight the need for integrated and transparent inventory systems.

A comparison of total MMR doses administered against the theoretical national target population revealed persistent misalignment. The IMSS and ISSSTE consistently reported coverage levels below the target throughout the study period, while the SSA reported over-application starting in 2012, potentially as a compensatory mechanism. Nevertheless, these efforts were insufficient to achieve optimal national coverage, which remained below the target in several years and exhibited substantial interannual variation. Notably, a marked increase in the reported coverage occurred between 2021 and 2024, although it is unclear whether this trend indicates genuine programmatic improvement or changes in reporting practices.

The evaluation of the %APP indicator for the MR vaccine confirmed inconsistent performance across institutions and years. In some years, more doses were administered than procured, while in others, fewer were applied. These fluctuations may partly be attributed to vaccine stock carryovers from previous years. However, in many cases, no plausible explanation could be identified, highlighting weaknesses in procurement and distribution oversight.

Persistently low coverage rates over time resulted in a substantial immunization gap. Between 2006 and 2024, an estimated 22.5 million MMR doses went unadministered, representing 26% of the total required to fully immunize the eligible population. This shortfall was particularly pronounced within the IMSS and ISSSTE systems, where coverage remained consistently below the target thresholds.

While the indicators of %PUR (percentage of doses procured), %APP (percentage of doses administered), and %COV (coverage) are widely used to assess immunization performance, their interpretation can be limited in fragmented healthcare systems. These indicators do not fully capture the complexity of decentralized vaccine delivery, inter-institutional variability, or local implementation gaps. Future assessments could benefit from developing integrated frameworks that combine spatial–temporal mapping, equity-adjusted coverage estimates, and indicators of system resilience. Such approaches would allow for a more nuanced evaluation of program performance in settings such as Mexico, where institutional responsibilities and population coverage are unevenly distributed.

The comparative analysis presented in Figure 8, supported by data from Table 1, further demonstrates discrepancies among the administrative data analyzed, ENSANUT surveys, and WHO-reported coverage for MCV1 (first dose of measles-containing vaccine) and MCV2 (second dose). The WHO estimates frequently exceeded 90%, with some years reporting values over 100%—particularly for MCV1—raising concerns about overestimation or misclassification of denominators. In contrast, ENSANUT and calculated institutional records reported lower more conservative coverage levels, ranging from 62% to 81% for MCV1. The divergences were particularly significant in the years following 2019, where the calculated coverage from this study fell substantially below the WHO-reported figures. While both WHO and national estimates reflect a decline in coverage during 2022–2024, the magnitude of these shifts varied considerably between sources.

WHO-reported MMR coverage rates are derived from administrative data submitted annually by national health authorities, where the numerator corresponds to the number of doses administered and the denominator represents the estimated target population of children eligible for vaccination. These data are reviewed, validated, and sometimes adjusted through WHO–UNICEF joint estimation processes to ensure international comparability. However, discrepancies may persist when national administrative figures differ from population-based survey estimates or when denominators are not updated according to demographic changes.

These inconsistencies highlight fundamental differences in estimation methodologies, data sources, and reporting systems. They also raise critical concerns about the accuracy of official coverage data, which are used to inform policy decisions and monitor progress toward regional measles and rubella elimination goals. Addressing these gaps will require improved integration of survey-based and administrative data systems, greater transparency in reporting practices, and the development of standardized indicators for more reliable monitoring of immunization performance.

The findings of this analysis align with those reported in other national studies, which have emphasized persistently low vaccination rates and considerable discrepancies between officially reported coverage figures and survey-based estimates. These disparities stem from differences in data sources, estimation methodologies, and reporting practices, along with the overestimation of official coverage or underreporting in national administrative data [15,16].

Other sources have pointed out the lack of reliable information needed to accurately assess true vaccination coverage levels in Mexico. Official data have consistently overestimated coverage, contributing to what Hernández-Avila [42] has termed a “false sense of security” regarding population-level immunity. This issue is corroborated by documented reports of critical vaccine shortages and substantial mismatches between administrative records and population-based surveys, as noted by Ríos-Blancas and colleagues [43]. These findings suggest that millions of children across the country may be missing full vaccination schedules or receiving them with significant delays.

This concern is further supported by seroepidemiological data. A national seroprevalence study conducted in 2022 revealed low measles-specific antibody prevalence among young adults aged 20 to 49 years, particularly among those born between 1989 and 2008 [44]. These findings likely reflect incomplete coverage of the second MMR dose and/or waning immunity in the absence of circulating virus during this period. Such susceptibility within this age group poses a significant public health risk, as it could facilitate the propagation of outbreaks in the event of measles importation. This risk has manifested: in the 42nd epidemiological week of 2025 (24 October 2025), Mexico reported 5019 confirmed measles cases and 23 deaths, with the most affected age group being 1 to 4 years, accounting for 14.5% (n = 726) of cases, followed by the 25- to 29-year-old group at 12.2% (n = 613) and those aged 30 to 34 years at 10.1% (n = 511). Regarding vaccination history, 91.7% (n = 4603) of cases had no documented record of prior vaccination during this outbreak [45].

Additionally, national surveillance data show a consistent annual burden of mumps cases, with notable increases observed in certain years, further reinforcing concerns about suboptimal immunization coverage and the potential for the resurgence of vaccine-preventable diseases. So far, no new cases of rubella have been reported, but ongoing vigilance is essential to maintain this status. Collectively, these findings emphasize the urgent need for evidence-based interventions to strengthen immunization systems, recover missed cohorts, and prevent future outbreaks.

Despite the high number of MR vaccine doses administered—over 62.4 million during the study period—evidence suggests that many individuals remain unprotected. This highlights the need to review current guidelines for MR vaccine administration and assess how the program is being implemented to ensure that doses are reaching those who need them.

The discrepancies observed between institutions can be traced to systemic fragmentation in procurement and governance processes. The institutional asymmetries reflect broader governance challenges, including the lack of a unified national forecasting mechanism, limited interoperability of information systems, and insufficient accountability in vaccine stock management. Similar governance-related inefficiencies have been documented in other decentralized health systems, where fragmentation and limited oversight undermine vaccine availability and timely administration [46,47].

The discrepancies identified in vaccine procurement and administration likely reflect broader systemic challenges. Potential contributing factors include weaknesses in cold-chain infrastructure, fragmented procurement processes, a lack of unified inventory tracking, and limited oversight in resource allocation and reporting. Similar patterns have been reported in other Latin American countries with decentralized healthcare systems, where institutional fragmentation and reduced transparency in management hinder effective vaccine delivery. Addressing these barriers is essential for improving program performance [43]. Furthermore, recent studies have highlighted how informal practices, opaque procurement procedures, and weak accountability mechanisms can exacerbate disparities in vaccine access and coverage, particularly in fragile healthcare systems [46,47]. These findings underscore the need for integrated monitoring tools and strong institutional governance to ensure reliable and equitable immunization delivery.

## 5. Conclusions and Recommendations

Despite challenges related to data availability and the scope of analysis, this study successfully evaluated the performance of Mexico’s national MMR vaccination program. By combining information from various institutions, estimating theoretical target populations, and using standardized coverage indicators, this study creates a strong basis for identifying systemic gaps and suggesting future improvements.

The analysis uncovered ongoing structural and operational challenges within Mexico’s MMR and MR vaccination programs over a 19-year period. It identified significant inconsistencies in procurement, administration, and coverage data across the country’s primary public health institutions. A notable issue was the mismatch between the number of vaccine doses acquired, the doses administered, and the estimated target population. Additionally, discrepancies among institutional, national, and international coverage reports point to limitations in data quality, transparency, and system integration. As a result, there is a significant immunization gap, especially among populations served by IMSS and ISSSTE, where millions of children may be missing complete or timely vaccination schedules.

Further complicating these issues, recent epidemiological and serological data highlight the real-world consequences of these gaps. The resurgence of measles in 2025 signifies the public health vulnerability stemming from years of insufficient or poorly coordinated immunization efforts. The consistently high number of mumps cases reported in recent years also emphasizes concerns about suboptimal MMR coverage.

To tackle these challenges and enhance national immunization efforts, the following recommendations are proposed: (1) Improve data integration and transparency by establishing a unified interoperable digital information system that consolidates procurement, administration, and population data across institutions, along with regular public reporting and independent audits. (2) Harmonize administrative and survey-based data through methodological alignment, clear definitions of denominators, and routine data reconciliation. (3) Implement predictive tools and contingency planning to prevent overstocking and shortages, ensuring efficient vaccine use and equitable distribution. (4) Launch targeted catch-up campaigns for at-risk groups, replacing the MR vaccine with the combined MMR vaccine to enhance protection against mumps. (5) Invest in routine serosurveillance, incorporating periodic representative seroprevalence studies into national monitoring systems to provide objective indicators of population immunity. (6) Adopt standardized tracking of nominal vaccination coverage across institutions, linked to national population registries to ensure accuracy and completeness.

Given the recent outbreaks and historical shortcomings, timely and coordinated action is crucial. Strengthening the MMR immunization program in Mexico will not only close existing immunity gaps but also protect the country’s long-standing achievements in elimination and bolster public confidence in vaccines.

## Figures and Tables

**Table 1:** Reported coverage of first and second doses of the MMR vaccine, Mexico, 2006–2023.

Year	2023		2022		2021		2020		2019		2018		2017		2016		2015	
Dose	1	2	1	2	1	2	1	2	1	2	1	2	1	2	1	2	1	2
WHO	76.4	73.7	82.5	85.8	103	97	83.3	104	73	73	97	99	62	76	98.3	96.5	100.9	96.1
EN *	71.3		61.8		72.6						64.8							
**Year**	**2014**		**2013**		**2012**		**2011**		**2010**		**2009**		**2008**		**2007**		**2006**	
Dose	1	2	1	2	1	2	1	2	1	2	1	2	1	2	1	2	1	2
WHO	98	96	88.62	75.7	98.8	91.8	98.4	96.6	91	95.2	92.1	96	94.3	95.5	85.3	96	96	55.2
EN *					81.2												78.4	

* National Health and Nutrition Survey (ENSANUT).

**Table 2:** Reported cases of rubella, mumps, and measles, Mexico, 2016–2025 [13].

Year	2025	2024	2023	2022	2021	2020	2019	2018	2017	2016
Rubella	0	0	0	0	0	0	0	0	0	20
Mumps	1040	3748	2027	2734	2301	3494	8009	8818	4519	3601
Measles	5053 [14]	0	0	0	0	196	1	5	0	0

**Table 3:** Target population for the first and second doses of MMR vaccination in Mexico, 2006–2024.

Year	2006	2007	2008	2009	2010	2011	2012	2013	2014	2015
12 months	2,299,151	2,298,258	2,307,115	2,319,419	2,329,577	2,329,754	2,315,845	2,288,969	2,258,535	2,235,734
6 years	2,334,716	2,342,092	2,333,358	2,313,057	2,296,540	2,289,510	2,290,080	2,255,036	2,305,548	2,311,233
Target	4,633,867	4,640,350	4,640,473	4,632,476	4,626,117	4,619,264	4,605,925	4,544,005	4,564,083	4,546,967
**Year**	**2015**	**2016**	**2017**	**2018**	**2019**	**2020**	2021 *	2022 *	2023 *	2024 *
12 months	2,235,734	2,214,966	2,186,443	2,142,836	2,112,354	2,109,981	2,098,926	2,091,094	2,083,347	2,050,080
6 years	2,311,233	2,306,483	2,287,586	2,259,450	2,227,893	2,206,235	2,188,443	2,163,565	2,122,316	2,093,173
Target	4,546,967	4,521,449	4,474,029	4,402,286	4,340,247	4,316,216	6,386,295	6,345,753	6,289,010	6,193,333

* The target population includes individuals aged 12 months, 18 months, and 6 years.

**Table 4:** Percentage of the population per institution per year (%).

	2006	2007	2008	2009	2010	2011	2012	2013	2014	2015	2016	2017	2018	2019	2020	2021	2022	2023	2024
IMSS	44	44	44	44	46	47	49	50	50	51	52	54	55	56	54	56	57	59	59
ISSSTE	10	10	10	10	10	11	11	11	11	11	11	11	11	11	11	11	11	11	11
Other	2	2	2	2	2	2	2	2	1	2	2	1	1	2	2	2	2	2	2
SSA	44	44	44	44	42	40	38	37	38	36	35	34	33	31	33	31	30	28	28

**Table 5:** Availability of vaccine procurement data by institution and vaccine (2006–2024).

Institution	MMR: Years Available	MMR: % Availability	MR: Years Available	MR: % Availability
SSA	9	47%	16	84%
IMSS	10	53%	9	47%
ISSSTE	15	79%	15	79%

## Data Availability

The data are available from sources of each institution cited in the references.
